# An Interactive E-learning Platform-Based Training to Improve Intensive Care Professionals' Knowledge Regarding Central Venous Catheter-Related Infections

**DOI:** 10.7759/cureus.46399

**Published:** 2023-10-03

**Authors:** Maria Foka, Theodoros Kyprianou, Nikolas Stylianides, Lefkios Paikousis, Lakis Palazis, Maria Kyranou, Elizabeth Papathanassoglou, Ekaterini Lambrinou

**Affiliations:** 1 Intensive Care Unit, Nicosia General Hospital, Nicosia, CYP; 2 Intensive Care and Emergency Medicine, University of Nicosia, Nicosia, CYP; 3 Respiratory and Intensive Care Medicine, King's College Hospital NHS Trust, London, GBR; 4 Electrical & Computer Engineering E-learning, Leafnet Ltd, Nicosia, CYP; 5 Statistician, Improvast, Nicosia, CYP; 6 Nursing, Cyprus University of Technology, Limassol, CYP; 7 Nursing, University of Alberta, Edmonton, CAN

**Keywords:** icu, distance education, continuing education, nurses, knowledge, intensive care unit, e-learning platform, cvc-related infections, central venous catheter

## Abstract

Introduction

The presence of a central venous catheter (CVC) leads to a high risk for blood infections, which are associated with increases in morbidity, mortality, and costs. This study aims to assess intensive care unit (ICU) nurses' and physicians' knowledge regarding the Centers for Disease Control and Prevention (CDC) guidelines for preventing CVC-related infections before and after an interactive distance education delivered through the e-learning platform Teleprometheus.

Materials and methods

The study was conducted among 85 nurses and physicians in Nicosia's General Hospital Intensive Care Unit (NGH-ICU) and high dependency unit (HDU). A validated questionnaire was used to assess nurses' and physicians' knowledge.

Results

Prior to the online interactive distance education, the mean total knowledge score was x̄ = 4.8 (SD = 2.46), while after, the mean total knowledge score increased to x̄ = 8.9 (SD = 2.38) (p<0.001). ICU physicians had a higher mean total knowledge score (x̄ = 10.20) than ICU nurses (x̄ = 8.75) after the intervention. There was no correlation between years of experience in the ICU and the level of knowledge (r = 0.048). The interactive distance education was positively evaluated by the participants, through a questionnaire, specially designed for this study.

Discussion

The most important findings were that (a) the level of knowledge of the participants improved with a statistically significant difference after the completion of the e-course, (b) the level of knowledge of the participants, after the completion of the e-course, was much higher from other studies, (c) there was no correlation between the years of experience of ICU health professionals and their level of knowledge, and (d) the interactive distance e-course was positively evaluated and satisfied the participants.

Conclusion

The current study demonstrates that in high-intensity work environments, such as ICUs, adopting e-learning approaches seems more necessary than ever.

## Introduction

Central venous catheters (CVCs) are the lifelines of the critically ill, as they enable the administration of life-saving medications, fluids, blood products, and parenteral nutrition [1]. At the same time, in situ CVCs are associated with a high risk of bloodstream infections that increase morbidity, mortality, and cost of care [2-4]. In 2011 (edited 2017), the Centers for Disease Control and Prevention (CDC) issued global recommendations on CVC care and the prevention of CVC-related infections [5]. However, their implementation is hampered by gaps in intensive care unit (ICU) professionals' knowledge and training [6-8]. Several studies, recently reviewed by our team [9], have shown that education and training programs can reduce the risk of CVC-related infections; however, the ICU environment poses significant, escalating, time- and physical presence-related educational constraints. E-learning and distance learning in various formats have been extensively used in healthcare training [10]. However, effectiveness data, particularly in demanding environments like ICUs, are scarce. This study aimed to assess the impact of an e-learning platform-based training program on ICU professionals' knowledge regarding preventing CVC-related infections. The primary outcome was the improvement in participants’ knowledge. The secondary outcomes were the exploration of the association between participants’ characteristics and the knowledge gained. Moreover, we explored the ICU professionals' satisfaction and the time they spent completing the interactive distance education.

## Materials and methods

Design

A prospective, quasi-experimental questionnaire-based study was conducted to assess the attitudes and theoretical knowledge of ICU physicians and nurses regarding the prevention of CVC-related infections before and after an e-learning platform facilitated educational intervention. Criteria for describing and evaluating training interventions in healthcare professions (CRE-DEPTH) guidelines were used for intervention developing and reporting [11].

Setting

The study was conducted in a twenty-three beds, multidisciplinary medical-surgical intensive care unit (ICU-17 beds) that supports critically ill patients and a high dependency unit (HDU-six beds) that supports level 2 ill patients, in a tertiary referral hospital.

Participants

As part of the process of checking the reliability of the questionnaire, a pilot study was conducted in which all health professionals (physicians and nurses) working in HDU were eligible to participate. HDU professionals were excluded from the interactive distance education to avoid bias. All the healthcare professionals (physicians and nurses) working in the ICU were eligible to participate in interactive distance education and answer the validated knowledge questionnaire.

Intervention

Selection, Translation and Intercultural Adaptation of the CVC Knowledge Questionnaire

To assess ICU professionals' attitudes and theoretical knowledge regarding the prevention of CVC-related infections, a Greek version of the questionnaire originally developed by Labeau et al. [12] and modified by Dedunska et al. [6] was used. The authors were contacted and written authorization for the translation and use of the questionnaire in the context of this study was obtained. The questionnaire assesses health professionals' knowledge of evidence-based guidelines for preventing infections associated with central venous catheters. It consists of two parts. Part one includes questions on demographic characteristics and other independent variables. Part two includes 11 multiple-choice questions. Correct answers were matched to recommendations issued by the CDC [5].

The English questionnaire was translated by two independent translators with a high level of language proficiency in both languages, who reside permanently in Cyprus and whose mother language is Greek. The first translator was not a health professional (GP), while the second was a health professional (ML) with experience in the subject matter of the questionnaire. In this way, errors are reduced and the quality of translation is improved. The different translations that emerged from the two translators were consolidated into a final form of the translated questionnaire in Greek. Any disagreements were discussed and resolved in the presence of the lead researcher [13]. The Greek questionnaire was translated back into English by two bilingual translators, who reside permanently in Cyprus and whose mother language is Greek (MC and VD). The researcher matched the "translations backward" with the standard foreign language questionnaire and noted the inconsistencies that existed. A review committee was set up to finalize the Greek version of the questionnaire. The committee consisted of (a) at least one bilingual member (VD), (b) a health professional (AP), and (c) translators who took part in the "forward translation" and "backward translation" (GP, Mc, Ml, VD). Disagreements were resolved, and the review committee concluded the first final version of the questionnaire in Greek. To obtain feedback regarding the question's clarity, a convenience sample of the Greek questionnaire's pre-final version was given to ten ICU professionals. The participants in the pilot study were selected by purposive sampling, so that the sample of the pilot study is representative of the target population in terms of as many characteristics as possible, such as gender, age, profession, and years of experience in the ICU. Finally, the final version was submitted to an expert committee, which, in consensus, established and confirmed the translation's semantic, idiomatic, experiential, and conceptual equivalence. As part of the process of checking the reliability of the questionnaire, a pilot study was conducted in which health professionals working in the HDU (a total of 18) participated. The questionnaire was given to the participants, and after a period of 30 days, it was given again. The period of 30 days was considered satisfactory to avoid the "memory effect", i.e., where the participants remembered the answers they gave when completing the first questionnaire. The HDU was chosen because the health professionals in this department treat patients with CVC lines. In addition, the HDU did not participate in the educational intervention later, so it did not affect the results of the questionnaire when it was administered to the target population at the ICU.

Interactive Distance Learning E-course

An interactive distance-learning e-course was developed. ICU health professionals' theoretical knowledge and attitudes were the targets of this program, facilitated by the e-learning platform previously developed and tested in the context of the EU-funded program Teleprometheus, a cross-border program between Greece and Cyprus for the period 2007-2013, co-funded by the EU and the Republic of Cyprus [14,15].

The interactive distance learning e-course was developed by adopting the basic principles of microlearning, an innovative pedagogy that refers to small lesson modules and short-term activities intended to teach and reinforce course objectives [16-18]. Furthermore, the e-course was developed by taking into consideration the basic principles of adult education [19,20]. One of the advantages is the asynchronistic aspect, which allows the participant to control the place, method, and time of access to information. Microlearning is also characterized by the remarkably quick delivery of educational content, within minutes or hours instead of days or months [16-18]. In our study, the material was delivered to participants in small learning "aliquots", using short videos, text, and images. In addition, the course was built in the form of an educational pathway [21-23], i.e., in very small sections, where participants should read the educational material, answer questions successfully, and then be allowed to proceed to the next section. Throughout the educational pathway, participants had the opportunity of continuous self-evaluation since, in case of an incorrect answer, the system led them again to study the relevant section. The e-learning platform was accessible from wherever there was an internet connection, e.g., from their home or work.

The content covered fifteen topics recommended by the Centers for Disease Control and Prevention (CDC) as best practices for the prevention of intravascular catheter-related infections [5]. These include (1) education, training, and staffing, (2) selection of catheter site, (3) hand hygiene and aseptic technique, (4) maximal sterile barrier precautions, (5) skin preparation, (6) catheter site dressing regimens, (7) patient cleansing, (8) catheter securement devices, (9) antimicrobial/antiseptic impregnated catheters and cuffs, (10) systemic antibiotic prophylaxis, (11) antibiotic/antiseptic ointments, (12) antibiotic lock prophylaxis, antimicrobial catheter flush and catheter lock prophylaxis, (13) replacement of CVCs, (14) replacement of administration sets, and finally (15) how to access the CVC line. Starting the interactive distance e-course, ICU health professionals were asked to answer the knowledge questionnaire. Up complication of education, they were asked to take the knowledge questionnaire again and also a questionnaire, specially designed for this study, to record their view of education and the time they have spent completing the interactive distance education.

Statistical analysis

Questionnaire

Test-retest reliability was conducted, which refers to the extent to which individuals' responses to the questionnaire items remain relatively consistent across repeated administrations of the same questionnaire. The agreement rate and Kappa index [24] between the response at the test and the response at the retest were investigated.

Educational Intervention Assessment

The Mann-Whitney U test statistical criterion was used to compare health professionals' level of knowledge before and after the intervention. A statistical comparison was made per statement with Fisher's exact test, comparing the frequencies of correct answers before and after the intervention. Statistical analysis was performed in R v.4.1.0 [25], and the Kappa index was estimated using the psych package [26]. Regarding the characteristics of the participants, a linear correlation coefficient of years of experience in the ICU was performed with the level of knowledge of the participants with the statistical criterion Pearson correlation coefficient. Finally, a statistical comparison of the improvement of knowledge after the intervention between doctors and nurses was performed with the statistical criterion t-test.

Ethical aspects

The study was reviewed and approved by the Cyprus National Bioethics Committee (approval number 2018.01.146) as well as the Ministry of Health Research Committee.

## Results

Knowledge questionnaire reliability check

In the statistical analysis of the questionnaire responses, Cronbach's alpha internal consistency index was a = 0.69 and was judged as satisfactory. The agreement rate and kappa index [24] between the responses to the test and the responses to the re-test were investigated. The kappa index and the agreement rate were high on all statements (>60%) (Table 1). The kappa values ​​were interpreted as follows: no correlation (0.00-0.20), satisfactory (0.21-0.40), moderate (0.41-0.60), strong (0.61-0.80), and almost perfect (0.81-1.00) [27].

**Table 1:** Kappa index and percentage of agreement in the test-retest for the scale (N = 16). CVC: central venous catheter. *Question 9 on the retest was answered incorrectly by all participants.

Questions	Kappa	Agreement rate
1. It is recommended to replace CVCs routinely	0.43	78%
2. In settings with a high rate of catheter-related infections, it is recommended to use a CVC coated or impregnated with an antiseptic agent	0.43	71%
3. It is recommended to change the clean, dry, and intact transparent dressing on the catheter insertion site	0.71	85%
4. It is recommended to change the clean, dry, and intact gauze dressing on the catheter insertion site	0.65	86%
5. It is recommended to cover up the catheter insertion site with	0.43	71%
6. It is recommended to disinfect the catheter insertion site with	0.51	78%
7. It is recommended to apply an antibiotic ointment at the insertion site of CVC	0.41	64%
8. When (1) lipid emulsions or (2) blood and blood products are administered through a CVC it is recommended to replace the administration set	0.51	79%
9. When liquids other than blood, blood products, or fat emulsions are administered continuously the administration set should be replaced	0*	7%
10. Administration sets used in intermitted infusion (when bottles with liquids are connected and disconnected for each dose) should be replaced	1	100%
11. It is recommended to use an antiseptic agent to clean the access hub or connector before the connection of the administration set or after unscrewing the dead-end cap to close on the catheter	0.19	64%

ICU healthcare professionals’ demographic characteristics

A total of 67 participants from the ICU took part in interactive distance education, of which 5 (7.5%) were physicians and 62 (92.5%) were nurses. The average experience in years of service was eight years (SD = 7), while the average experience in years in this ICU was 5.1 years (SD = 5). Thirty-five hold a bachelor's degree (52%), 30 (45%) a master's degree, and two (3%) a doctoral degree (Table 2). About 48/67 participants (71.6%) stated that they had received some kind of education, training, or information about CVC maintenance, before the intervention (Table 2).

**Table 2:** Demographic characteristics of participants (Ν = 67).

Variable	Years	%
Age	34 (SD = 7)	
Sex		
Male		21 (31%)
Female		46 (69%)
Years of practice	8 (SD = 7)	
Years of practice in the ICU	5.1 (SD = 5)	
Profession		
Physicians		5 (7.5%)
Nurses		62 (93%)
Education		
Bachelor’s degree		35 (52%)
Master’s degree		30 (45%)
PhD degree		2 (3%)

The majority of them (N = 29/48-50.9%) received clinical bedside demonstrations in CVC maintenance (Table 3).

**Table 3:** Previous education on CVC maintenance. CVC: central venous catheter.

Variables	N	%
Clinical demonstration bedside	29	50.9%
Presentation	23	40.4%
Clinical guidelines reading	20	35.1%
Educational video	14	24.6%
Seminar or course	3	5.3%
Other	13	22.8%

Effect of intervention on ICU healthcare professionals' knowledge

Fifty-eight out of a total of 67 participants who registered for the interactive distance education have completed it (nine ICU nurses did not). Before the education, the average knowledge value was x̄ = 4.8 (SD = 2.46), while after education, the average knowledge value increased to x̄ = 8.9 (SD = 2.38) (Mann-Whitney U test, p<0.001) (Table 4).

**Table 4:** Effect of interactive distance education on ICU professionals’ knowledge (Ν = 58). *Mann-Whitney U test.

Variable	Before the intervention, x̄ (SD) N = 58	After the intervention, x̄ (SD) N = 58	P*
Knowledge score (mean (SD))	4.84 (2.46)	8.88 (2.38)	<0.001

**Figure 1:**
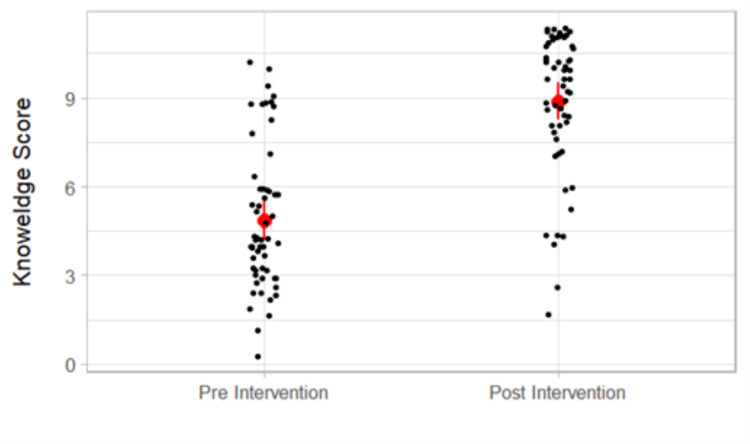
Participants' performance before and after interactive distance learning.

Participants' performance before and after interactive distance education is also graphically shown in Figure 1.

Table 5 shows the number (%) of participants who noted the correct answer to each statement of the knowledge tool. All questions improved their success rate after interactive distance education. Statistically significant improvement (p<0.05) after the intervention was observed in all statements except statement two (p = 0.15) and statements four and 10, in which the statistical improvement was marginal (p = 0.5). In addition, Table 5 shows the distribution of the correct answers to each statement and at each time point (before and after the education).

**Table 5:** The number (%) of those who noted the correct answer before and after the intervention (N = 58). CVC: central venous catheter. † "Chisq.test" for categorical variables with all expected cell counts ≥5, and "fisher.test" for categorical variables with any expected cell count <5. *Correct answer.

Questions	Before	After	p†
It is recommended to replace CVCs routinely			0.001
Yes, every three days	2 (3.4%)	0 (0%)	
Yes, every seventh day	21 (36%)	7 (12%)	
No, only when indicated*	35 (60%)	51 (88%)	
In settings with a high rate of catheter-related infections, it is recommended to use a CVC coated or impregnated with an antiseptic agent			0.12
Yes, in patients whose CVC is expected to be remained in place for >5 days*	44 (76%)	51 (88%)	
No, because the use of such catheters is not cost-effective	0 (0%)	0 (0%)	
No, because the use of such catheters does not result in a significant decrease in the rate of catheter-related infections	8 (14%)	6 (10%)	
I do not know	6 (10%)	1 (1.7%)	
It is recommended to change the clean, dry, and intact transparent dressing on the catheter insertion site			<0.001
Every two days	40 (69%)	7 (12%)	
Every fifth day	3 (5.2%)	5(8.6%)	
Every seventh day*	14 (24%)	44 (76%)	
Only the catheter is replaced	1 (1.7%)	2 (3.4%)	
It is recommended to change the clean, dry, and intact gauze dressing on the catheter insertion site			0.036
Every two days*	44 (77%)	48 (83%)	
Every fifth day	0 (0%)	2 (3.4%)	
Every seventh day	5 (8.8%)	7 (12%)	
Only the catheter is replaced	8 (14%)	1 (1.7%)	
(No response)	1	0	
It is recommended to cover up the catheter insertion site with			<0.001
Polyurethane dressing (transparent, semipermeable)	37 (64%)	14 (24%)	
Gauze dressing	0 (0%)	1 (1.7%)	
Both are recommended because they do not affect the risk for catheter-related infections*	21 (36%)	43 (74%)	
I do not know	0 (0%)	0 (0%)	
It is recommended to disinfect the catheter insertion site with			<0.001
2% Aqueous chlorhexidine	43 (74%)	14 (24%)	
0.5%-2% Alcoholic gluconate chlorhexidine*	13 (22%)	44 (76%)	
10% Povidone-iodine	1 (1.7%)	0 (0%)	
I do not know	1 (1.7%)	0 (0%)	
It is recommended to apply an antibiotic ointment at the insertion site of the CVC			<0.001
Yes, because it decreases the risk for catheter-related infections	3 (5.2%)	2 (3.4%)	
No, because it causes antibiotic resistance*	16 (28%)	49 (84%)	
No, because it does not decrease the risk for catheter-related infections	31 (53%)	6 (10%)	
I do not know	8 (14%)	1 (1.7%)	
When (1) lipid emulsions or (2) blood and blood products are administered through a CVC, it is recommended to replace the administration set			0.001
Every 12 h, four blood units or blood components can be administered through one administration set	21 (36%)	8 (14%)	
Within 24 h, one blood unit or blood component can be administered through one administration set*	27 (47%)	47 (81%)	
Every 72 h, two blood units or blood components can be administered through one administration set	7 (12%)	2 (3.4%)	
I do not know	3 (5.2%)	1 (1.7%)	
When liquids other than blood, blood products, or fat emulsions are administered continuously the administration set should be replaced			<0.001
Every 48 h	24 (41%)	4 (6.9%)	
Every 72-96 h	26 (45%)	14 (24%)	
Every four to seven days*	8 (14%)	40 (69%)	
Administration sets used in intermitted infusion (when bottles with liquids are connected and disconnected for each dose) should be replaced			0.2
Every 24 h*	41 (71%)	45 (78%)	
Every 72 h	10 (17%)	7 (12%)	
Every 96 h	1 (1.7%)	4 (6.9%)	
I do not know	6 (10%)	2 (3.4%)	
It is recommended to use an antiseptic agent to clean the access hub or connector before the connection of the administration set or after unscrewing the dead-end cap closes the catheter			<0.001
Yes, by wiping with 70% alcohol solution or alcohol and chlorhexidine solution for no less than 15 s*	18 (31%)	53 (91%)	
Yes, by spraying the access site with 70% alcohol solution or alcohol chlorhexidine solution	39 (67%)	5 (8.6%)	
It is not recommended because no evidence has been found for the relation between the disinfections of the connecting site of the administration set and the contamination of fluids or the insertion hub	0 (0%)	0 (0%)	
I do not know	1 (1.7%)	0 (0%)	

ICU experience and level of knowledge correlation

**Figure 2:**
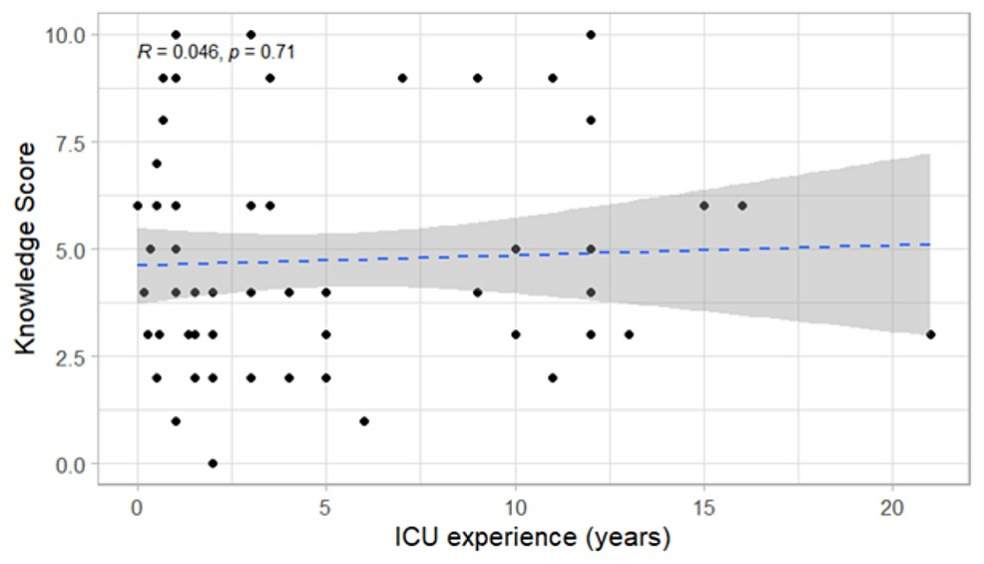
Scatterplot for the correlation of experience in ICU with the level of knowledge. ICU: intensive care unit.

There was no correlation between the years of experience of ICU health professionals and their level of knowledge (r = 0.048) (Figure 2).

Differences between nurses and physicians 

Only five physicians participated in the intervention, and they showed a higher average value of knowledge compared to nurses. The improvement in the mean value of knowledge in physicians was x̄ = 4.2 (SD = 0.84), and the improvement in the mean value of knowledge in nurses was x̄ = 4.02 (SD = 2.99). There was no difference in the improvement they had after the training (p>0.9) (Table 6).

**Table 6:** Nurses’ and physicians’ knowledge before and after education.

Time	Physicians x̄ (SD) N = 5	Nurses x̄ (SD) N = 54	p
Before intervention	6.00 (0.00)	4.74 (2.54)	0.068
After intervention	10.20 (0.84)	8.75 (2.44)	0.2
Difference	4.20 (0.84)	4.02 (2.99)	>0.9

Health professionals' views and experiences of interactive distance e-course

Forty-two out of a total of 58 participants who completed the e-course answered the questionnaire, which captured their opinion about the interactive distance e-course. The questionnaire consisted of a total of eight statements, of which the first two recorded the time taken by the participants to complete the e-course and how many times they entered to complete it (statements 1 and 2), and six statements expressed their view on the interactive distance e-course (statements 3-8). Table 7 presents in detail the answers of the participants.

**Table 7:** Completion time (statements 1 and 2) and view of participants (statements 3 to 8) from the interactive distance education (N = 42). *: n (%)

Questions	N = 42*
1. How much time did you spend completing the e-course	
0-2 hours	37 (88%)
2-4 hours	3 (7.1%)
4-6 hours	2 (4.8%)
2. How many times did you enter the e-course to be able to complete it	
One time	28 (67%)
Two times	10 (24%)
Three to four times	4 (9.5%)
3. How was the tour in the e-course	
Very easy/easy	30 (71%)
Neutral	12 (29%)
Difficult/very difficult	0 (0%)
4. The structure of the e-course was presented in an organized way	
Strongly agree/agree	38 (90%)
Neither agree or disagree	3 (7.1%)
Disagree/strongly disagree	1 (2.4%)
5. Completing the e-course was easy for me	
Strongly agree/agree	39 (93%)
Neither agree or disagree	2 (4.8%)
Disagree/strongly disagree	1 (2.4%)
6. I would encourage other colleagues to attend this e-course	
Strongly agree/agree	39 (93%)
Neither agree or disagree	1 (2.4%)
Disagree/strongly disagree	2 (4.8%)
7. The level of difficulty of the e-course was	
Very easy/easy	21 (50%)
Neutral	19 (45%)
Difficult/very difficult	2 (4.8%)
8. My overall assessment of the usefulness of this e-course is	
1-3	6 (14%)
4	10 (24%)
5	26 (62%)

To check whether the participants answered the questionnaire in a way that confirmed the reliability of their answers, Cronbach's alpha internal consistency index was checked in the six statements that expressed their views about the e-course (statements 3-8). The six statements that recorded their view on education were: statement 3 (the tour of the e-course is), statement 4 (the structure of the e-course was presented in an organized way), statement 5 (the completion of the e-course was easy for me), statement 6 (I would encourage other colleagues to attend this e-course), statement 7 (the level of difficulty of the e-course was), and statement 8 (my overall assessment of the usefulness of this e-course is). The six statements showed Cronbach's alpha: 0.85. In addition, the correlation of the six statements of satisfaction (statements 3-8) was studied, with the time that the participants stated that they devoted to the completion of the course, using the calculation of the average score in each statement and comparison with the parameter of the completion time of the course. The answers to the six statements were numerically coded as follows: strongly agree = 5, agree = 4, neither agree nor disagree = 3, disagree = 2, strongly disagree = 1. The purpose of the coding was to calculate the average satisfaction in each statement and compare it with the parameter of the completion time of the course. Table 8 shows the average value in each application, depending on the time of completion of the course. It was observed that the participants who devoted only 0-2 hours were more satisfied with statement 4 (the structure of the e-course was presented in an organized way), statement 6 (I would encourage other colleagues to attend this e-course), and statement 5 (completing the e-course was easy for me) than the participants who completed the course in two to four hours or four to six hours.

**Table 8:** Comparison of completion time with an average value of x̄ in the six statements. 1. Mean (SD). 2. One-way ANOVA.

Statements	0-2 hours x̄ (SD) N = 37^1^	2-4 hours x̄ (SD) N = 3^1^	4-6 hours x̄ (SD) N = 2^1^	p-value^2^
Statement 3. How was the tour in the e-course	4.2 (0.8)	3.7 (1.2)	3.0 (0.0)	0.085
Statement 4. The structure of the e-course was presented in an organized way	4.5 (0.6)	3.7 (0.6)	2.5 (0.7)	<0.001
Statement 5. Completing the e-course was easy for me	4.5 (0.6)	3.3 (1.2)	3.5 (0.7)	0.002
Statement 6. I would encourage other colleagues to attend this e-course	4.7 (0.5)	3.7 (1.5)	2.5 (0.7)	<0.001
Statement 7. The level of difficulty of the e-course	3.8 (0.9)	4.0 (1.0)	2.5 (0.7)	0.15
Statement 8. My overall assessment of the usefulness of this e-course	4.5 (0.9)	4.0 (1.0)	4.0 (0.0)	0.63

## Discussion

Based on previous findings [9] and taking into consideration the basic principles of adult education [19,20] and new methods of building and delivering education as a microlearning and educational pathway [16-18,21-23], the research team decided to develop and deliver an interactive distance e-course and studied the effect on the level of knowledge of ICU health care professionals about clinical guidelines for the prevention of bloodstream CVC-related infections. The most important findings were that (a) the level of knowledge of the participants was improved with a statistically significant difference after the completion of the e-course, (b) the level of knowledge of the participants, after the completion of the e-course, was much higher from other studies [6-8,12], (c) there was no correlation between the years of experience of ICU health professionals and their level of knowledge, and (d) the interactive distance e-course was positively evaluated and satisfied the participants.

ICU healthcare professionals’ demographic characteristics

In the present study, the mean age of the participants was 34 years old, while the mean age of the participants in other studies that used the same knowledge questionnaire was higher [6,7,28]. In addition, the percentage of men in the ICU conducting the study was 31%, similar to another two studies [12,28]. On the contrary, two studies from Poland show that the percentage of men in the ICU was under 5% [6,7], and one study from Jordan shows that the percentage of men working in the ICU was 58% [8]. The average working experience in the ICU in the present study was 5.1 years, and it was similar to other studies that used the same knowledge questionnaire [8,12,28]. On the contrary, two studies from Poland show that the average working experience in the ICU was above 15 years [6,7]. 

ICU professionals’ knowledge of the guidelines for preventing CVC-related infections before and after interactive distance education

In the present study, before the interactive distance education, participants showed a higher level of knowledge (x̄ = 4.8) compared to the results in the Labeau et al. study [12], in which the mean value was x̄ = 4.4, and in the Al Qadire et al. study [8], in which the mean value for nurses was x̄ = 3.3 and for physicians x̄ = 2.6. In contrast, in the study by Friedt et al. [28], the mean value was x̄ = 5.7, i.e., higher than the mean value in the present study. The worst-answered question before the interactive distance education was question number nine regarding the replacement of the devices, in the case of continuous intravenous infusion of fluids and drugs. Guidelines recommend their replacement not more often than 96 hours, but at least every seven days [5]. Only 14% of the participants, in the present study, answered correctly. In the Dedunska et al. study [6], only 6% of the participants knew the correct answer. In the Al Qadire et al. study [8], 17% of nurses and 17% of physicians answered correctly. Dedunska et al. [6] interpreted the large percentage of incorrect answers in the unclear separation of (a) continuous infusion devices and (b) intermitted infusion devices. It is possible that the unclear separation also affected healthcare professionals' answers in the present study. Furthermore, question number six was answered wrong by a large percentage of participants in the present study before the interactive distance education. CDC Guidelines recommend the use of 0.5-2% alcoholic gluconate chlorhexidine to disinfect the CVC insertion site [5]. As a second alternative, the use of povidone-iodine or 70% alcohol is recommended [5]. Only 22% of the participants in the present study answered correctly, while 74% incorrectly answered that the use of simple chlorhexidine 2% is recommended. About 2% alcoholic gluconate chlorhexidine was used in the ICU of the study in 2013; however, healthcare professionals referred to it as "chlorhexidine." In the Dedunska et al. study [6], 49% of the participants knew the correct answer, and in the Dyk et al. study [7], 57% of the participants knew the correct answer. In the Al Qadire et al. study [8], only 29% of nurses and 14% of doctors answered correctly.

After attending the interactive distance education, the participants increased their mean value of knowledge (x̄ = 8.9, p<0.001). Participants showed an improvement in their knowledge on all 11 questions of the knowledge questionnaire. In eight of the 11 questions, a statistically significant difference was recorded at p<0.001 (questions 1, 3, 5, 7, 8, 9, and 11), and in the two marginally statistically significant differences, p = 0.05 (questions 4 and 10) were established. The only question that did not show a statistically significant difference was question number two, which stated that in settings with a high rate of CVC-related bloodstream infections, it is recommended to use a CVC coated or impregnated with an antiseptic agent, in which participants answered largely correct before (76%) and after (88%) interactive distance education.

ICU healthcare’s knowledge level

ICU physicians in the present study, had a higher mean total knowledge score than ICU nurses, before and after the interactive distance education (Table 6). ICU physicians' and nurses' knowledge level, in the present study, is higher, even before the interactive distance education, compared to the mean total knowledge score of ICU physicians and nurses in the Jordan study [8] and in the 19 European ICUs study [12]. This is probably because in the ICU where this study was conducted, local training in CVC placement and maintenance has been offered in recent years, and 71% of the participants stated that they had the opportunity to attend some form of in-house training, and at least 50% of the participants stated that they have received clinical training in the ICU. On the contrary, ICU physicians in the study from Jordan reported that only 12% had received any training in CVC placement or maintenance [8]. Al Qadire et al. [8] emphasized the need for continuing education, personnel knowledge evaluation, and supervision of the implementation of clinical guidelines in daily clinical practice. On the contrary, Dyk et al. stated that nurses who have had the opportunity to attend some form of education presented a statistically significant difference in their level of knowledge compared to nurses who reported that they did not attend any training [7].

After the interactive distance education, ICU health professionals, in the present study, showed a higher knowledge level from all the studies that they used the same knowledge questionnaire. It appeared that there is no correlation between ICU experience (years) and the level of knowledge. Similar results were seen in both studies on ICU nurses in Poland, where the comparison of ICU experience (years) with nurses' level of knowledge did not show a statistically significant difference [6,7]. This result reinforces the view that it does not matter how many years one works in an intensive care unit, but how often one is trained to be able to present a high level of knowledge.

Health professionals view for interactive distance e-courses and the time they spent to complete the education

With the completion of the interactive distance e-course, participants were asked to answer a questionnaire, specially designed for this study, which recorded their view of education and the time they had spent (Table 7). In statement 1 (how much time you spent in total to complete the course), 88% said it took less than two hours. This finding is extremely important and confirms that interactive distance education has been delivered in an extremely short time. A total of 20 modules of educational material (information material, educational material, self-assessment per module, and knowledge assessment questionnaire before and after the course) were delivered to the participants in less than 120 minutes. The structure of educational material, adopting (a) the basic principles of adult education [19,20], (b) microlearning [16-18], and (c) the educational pathway [21-23], seems to have yielded a positive result, as participants stated positive view in all eight statements (Table 7). Participants had the opportunity to stop the e-course at any point they wished and continue later. About 67% of the participants completed the e-course by entering only once. One possible interpretation is that the e-course kept the interest of the participants alive to such an extent that a large percentage of participants chose to start and finish the e-course without disconnecting even once. The overall assessment of the usefulness of the e-course they attended was rated as very good and/or excellent by 86% of the participants (Table 7). In conclusion, the research team has created an interactive distance education, which was positively evaluated and satisfied the participants, while at the same time achieving its goal, which was to improve the level of knowledge of health professionals working in the ICU regarding the guidelines for preventing CVC-related infections.

Add to the literature

In high-intensity work environments, such as ICUs, it is difficult to implement traditional educational programs for health professionals only during working hours. This argument was further proven during the COVID-19 pandemic. Adopting innovative solutions, such as integrating educational platforms, that can be accessed even through smart mobile phones, seems more necessary today than ever. In the present study, the Tele-Prometheus platform was used, and a short e-course was delivered based on new technologies. Participants spend only two hours completing the education program with excellent results without spending working hours, therefore adding more organization costs.

Study limitations

The present study is a single-center study and was limited only to one ICU; thus, the results can’t be generalized and be indicated for the country. Furthermore, the small ICU physicians’ population prevents the study of variables that influence physicians' knowledge.

## Conclusions

The current study demonstrated that nurses and physicians had a high level of knowledge, compared to previous studies, regarding the CDC guidelines for preventing CVC-related infections before education. It is very important, though, that even starting from a higher level of pre-intervention knowledge, significant improvement can be observed using an e-learning platform-based, unsupervised training program. Our results showed that in high-intensity work environments, such as ICUs, adopting innovative solutions seems more necessary today than ever.
